# Characterisation and use of a functional *Gadd45g* bacterial artificial chromosome

**DOI:** 10.1038/s41598-018-35458-5

**Published:** 2018-11-23

**Authors:** Nick Warr, Joel May, Lydia Teboul, Toru Suzuki, Maki Asami, Anthony C. F. Perry, Sara Wells, Andy Greenfield

**Affiliations:** 1 Mammalian Genetics Unit, MRC Harwell Institute, Oxfordshire, OX11 0RD UK; 2 The Mary Lyon Centre, MRC Harwell Institute, Oxfordshire, OX11 0RD UK; 3 Laboratory of Mammalian Molecular Embryology, Department of Biology and Biochemistry, University of Bath, Bath, BA2 7AY UK; 4 Present Address: Boston University School of Medicine, Vascular Biology Section, 650 Albany St, X720, Boston, MA 02118 USA

## Abstract

Bacterial artificial chromosomes (BACs) offer a means of manipulating gene expression and tagging gene products in the mammalian genome without the need to alter endogenous gene structure and risk deleterious phenotypic consequences. However, for a BAC clone to be useful for such purposes it must be shown to contain all the regulatory elements required for normal gene expression and allow phenotypic rescue in the absence of an endogenous gene. Here, we report identification of a functional BAC containing *Gadd45g*, a gene implicated in DNA repair, DNA demethylation and testis determination in mice and exhibiting a broad pattern of embryonic expression. Mouse fetuses lacking the endogenous *Gadd45g* gene undergo normal testis development in the presence of the *Gadd45g* BAC transgene. Moreover, a survey of embryonic *Gadd45g* expression from the BAC reveals that all reported sites of expression are maintained. This functional BAC can now be used for subsequent manipulation of the *Gadd45g* gene with the confidence that regulatory elements required for embryonic expression, including testis determination, are present. We describe the generation and characterisation of a *Gadd45g*-mCherry fluorescent reporter exhibiting strong expression in developing gonads and neural tissue, recapitulating endogenous gene expression, as evidence of this.

## Introduction

GADD45γ is a member of a family of proteins (Growth arrest and DNA damage response) implicated in DNA repair and active DNA demethylation^1,2^, and the activation of mitogen activated protein kinase (MAPK) signalling^3^. Whilst initially thought to be dispensable for mouse development^4^, loss of function studies reveal a role for GADD45γ in fetal testis determination via its positive effects on p38 MAPK and *Sry* expression^5–7^. In the adult, GADD45γ has reported roles in cardiomyocyte apoptosis following myocardial infarction^8^, and in regulating the thermogenic capacity of brown adipose tissue^9^. Careful profiling of expression in the developing cerebral cortex also suggests roles for *Gadd45a*, *Gadd45b* and *Gadd45g* in neurogenesis^10^. However, the precise molecular roles played by GADD45γ in these different physiological contexts remain to be elucidated.

The study of gene function can be greatly enhanced by the use of fluorescent reporters that allow live imaging of cellular processes. A previous attempt at generating a reporter for *Gadd45g* used 1767 base-pairs (bp) of DNA immediately upstream of the gene’s transcription start-site to drive Venus expression in mice^11^. Whilst reporter expression was clearly detectable in neuronal cell-types, there was no expression detected in the developing gonads (ref.^11^ and Takumi Kawaue, pers. comm.). Fetal gonadal expression of *Gadd45g* underlies its role in testis determination and the absence of gonadal expression in these transgenic mice suggests that enhancers required for gonadal expression are not found within this 1767 bp region.

Introducing reporters or tags into endogenous genes has been made easier by genome editing technology, but any such alterations risk disrupting the function of a gene, especially when larger sections of heterologous DNA are introduced. This may result in haploinsufficiency and unwanted phenotypic effects. An alternative approach to ensuring that all regulatory elements required for expression are present is the use of large bacterial artificial chromosomes (BACs), which contain a gene of interest and commonly large regions of adjacent DNA (up to 500 kb)^12^. Transgenic mice can be readily made using microinjection of BAC DNA into 1-cell embryos^13^ or into metaphase II (MII) oocytes, at the same time as intracytoplasmic sperm injection (ICSI)^14^. Moreover, recombineering technology can be used to modify the gene carried by the BAC and thereby permit study of, amongst other things, gene expression and protein localisation^15^. The latter is particularly useful if a good antibody is not available. But prior evidence is required that expression from the BAC recapitulates the endogenous gene.

Here we characterise a novel mouse line carrying a *Gadd45g* BAC transgene. This transgene allows normal sexual development in the absence of the endogenous gene. Moreover, careful examination of expression from the BAC indicates that both gonadal and broader fetal expression matches that of the endogenous gene. This BAC clone is appropriate for modification to further explore the developmental roles of *Gadd45g* and may be suitable for investigations of adult function. Finally, we report the generation of a *Gadd45g*-mCherry reporter that can be used for a variety of imaging applications.

## Results

**Figure 1:**
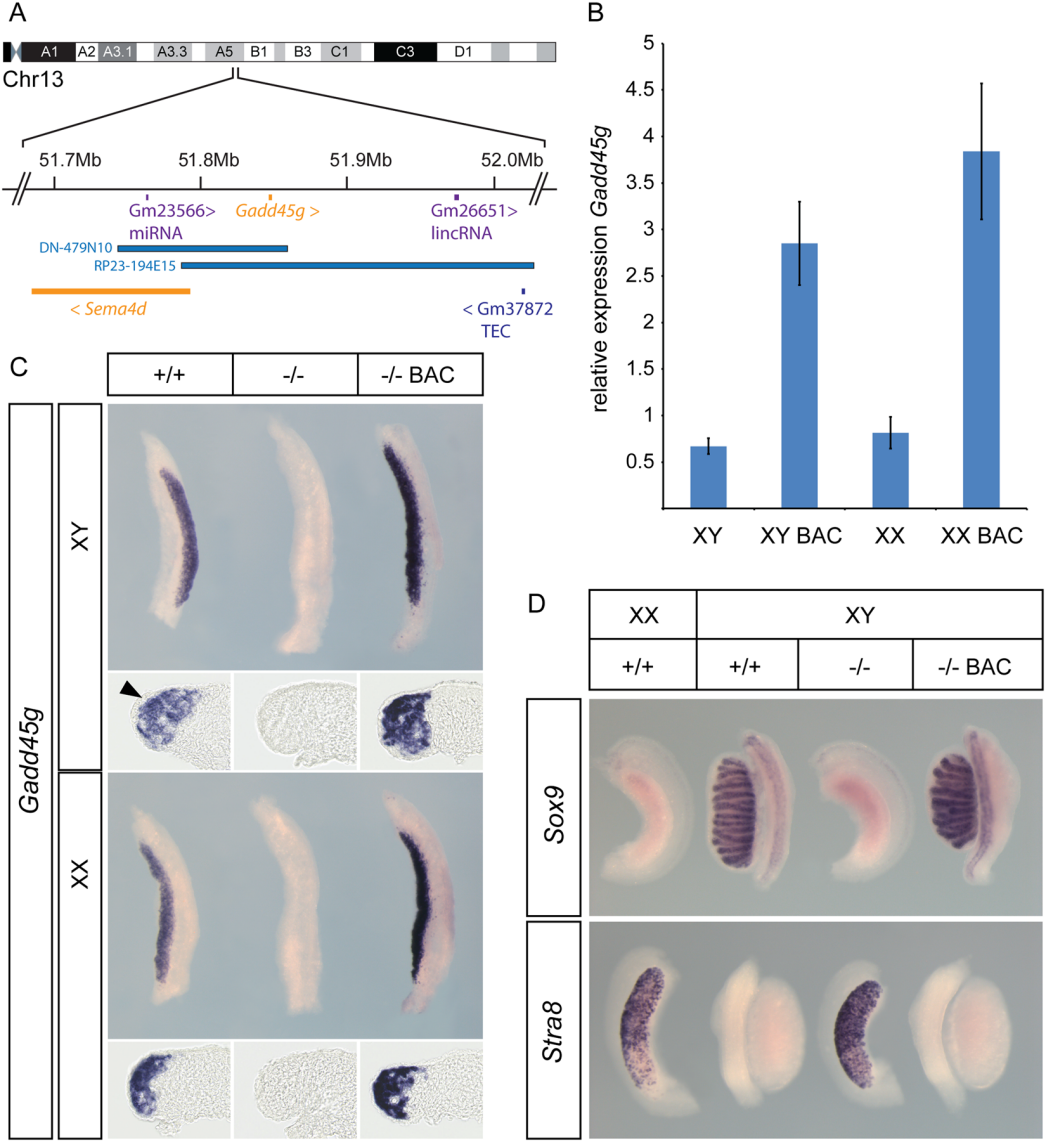
(**A**) Position of BACs on chromosome 13 containing *Gadd45g*; (**B**) qRT-PCR reveals elevated expression of *Gadd45g* in transgenic (XY BAC, XX BAC) gonads at 11.5 dpc (18 ts) compared to XY and XX wild-type controls; (**C**) WMISH of *Gadd45g* in XY and XX gonads from wild-type (+/+), null (−/−) and E15 BAC transgenic null (−/− BAC) embryos at 11.5 dpc. Transverse sectioning (lower panel) reveals that expression is absent from the coelomic epithelium (arrow head); (**D**) The presence of the E15 BAC rescues XY gonadal sex reversal caused by absence of endogenous *Gadd45g* (−/−) based on *Sox9* (Sertoli cell) and *Stra8* (meiotic entry germ cells) marker expression.

To identify BAC clones containing *Gadd45g* we searched *Ensembl* and identified two potential candidates (Fig. 1A). We selected clone RP23–194E15 (henceforth E15), a 238.3 kb BAC, on the basis of the large amount of DNA flanking the *Gadd45g* gene on both sides: 59.1 kb 5′ of the gene and 177.4 kb 3′ of the gene. E15 DNA was prepared and a PCR analysis was performed to ensure that all exons were present. Coding regions were sequenced to determine whether any mutations existed. Once it had passed this quality control, DNA was used to inject 1-cell mouse embryos and generate transgenic founders. Five founder mice were found to contain E15 BAC vector sequences. These founders were bred to check whether the transgene was transmitted to offspring. Four founders were found to transmit. All studies below refer to a line derived from founder 5 (line 5). This was the only line found to be capable of phenotypic rescue in experiments described below.

We first determined whether embryos transgenic for E15 had elevated levels of *Gadd45g* in the gonad at the stage of sex determination (11.5 dpc). Quantitative reverse-transcription PCR (qRT-PCR) revealed that transgenic gonads had approximately 5-fold higher levels of *Gadd45g* (Fig. 1B). These data were consistent with wholemount *in situ* hybridisation (WMISH) showing that *Gadd45g* expression in XY and XX gonads at this stage was stronger in homozygous *Gadd45g* knockout embryos when the transgene was present than in wild-type controls (Fig. 1C). No expression was detected in homozygotes in the absence of the transgene. Expression was not detected in the coelomic epithelium, as previously reported^6^.

Given this gonadal expression, we then tested whether the presence of the E15 transgene could rescue testis determination defects in XY *Gadd45g* homozygous knockouts. In the absence of *Gadd45g*, XY gonads have an ovarian morphology, lack *Sox9* and express *Stra8*, a marker of germ cell meiotic entry normally restricted to the ovary (Fig. 1D). In contrast, transgenic embryos that lacked the endogenous gene formed testes like XY wild-type controls (Fig. 1D). These had high levels of *Sox9* in testis cords and no detectable *Stra8* expression. Thus, rescue of the sex reversal phenotype is complete in the presence of the E15 transgene.

**Figure 2:**
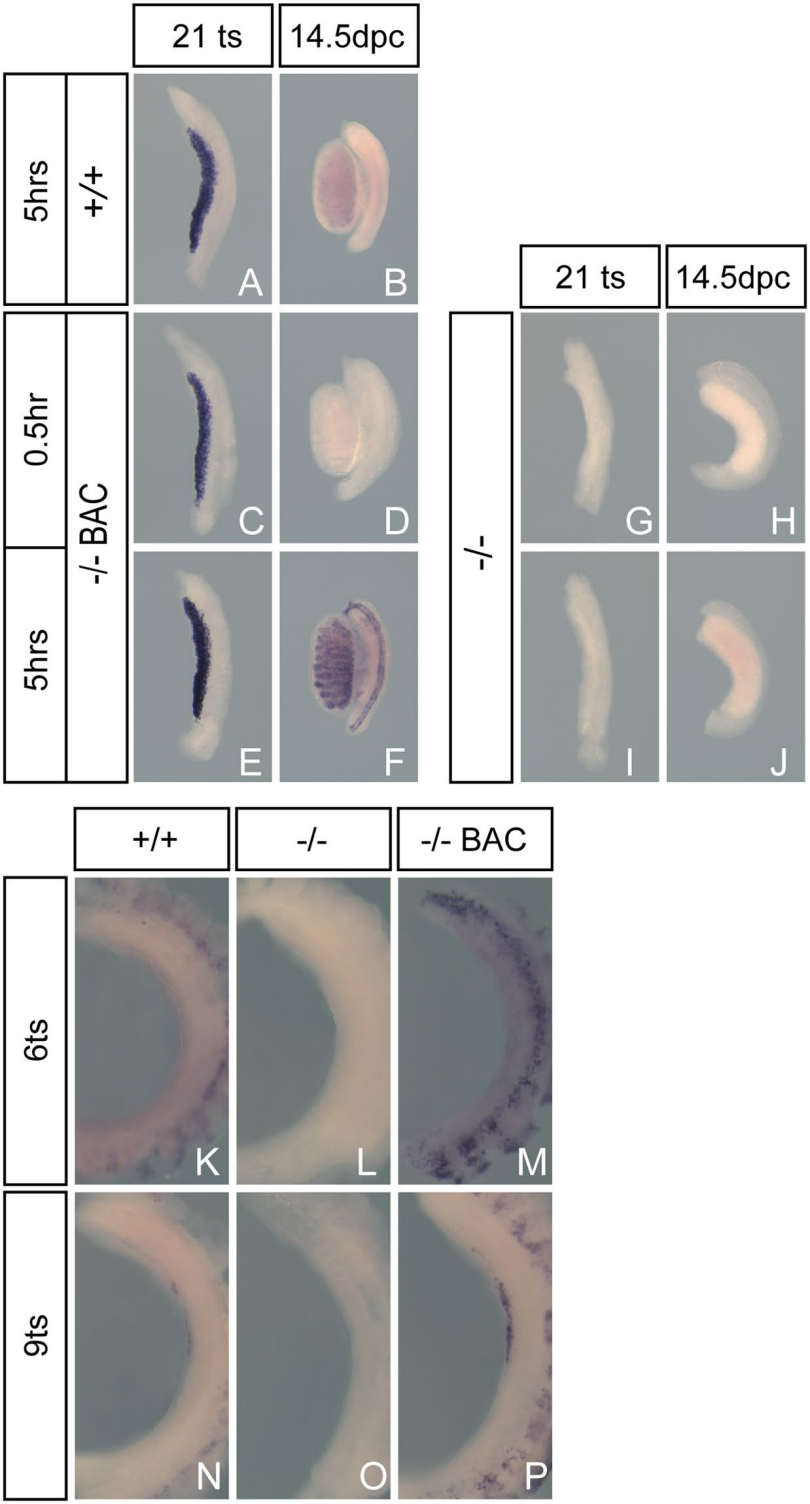
WMISH analysis of *Gadd45g* expression in XY wild-type (+/+) at 21 ts (**A**) and 14.5 dpc (**B**). Expression is also shown for transgenic null (−/− BAC) gonads at 21 ts following staining for 0.5 hours (**C**) or 5 hours (**E**) and similarly at 14.5 dpc (**D**,**F**). No expression is detectable in null (−/−) gonads at any stage or length of staining (**G**–**J**). No *Gadd45g* expression is seen at 6ts in embryos of any genotype (**K**–**M**). Expression is observed at 9 ts in wild-type (**N**) and more strongly in transgenic null embryos (**P**) but not in null embryos (**O**).

Given this functional rescue, we sought to determine whether the onset and cessation of the BAC-encoded *Gadd45g* was comparable to that of the endogenous gene. This is still important to test and will determine whether this BAC is suitable for studying the temporal dynamics and cellular specificity of *Gadd45g* expression. We performed WMISH analysis on XY samples at 11.75 dpc (21 tail-somites (ts) stage) and 14.5 dpc using two distinct lengths of time for staining to develop: short (0.5 hrs) and long (5 hours). Wild-type samples required the longer staining time to allow signal to be reliably detected (Fig. 2). This revealed strong expression at 11.5 dpc but no detectable signal at 14.5 dpc (Fig. 2A,B), consistent with previous reports^5–7^. In contrast, expression in transgenic 11.5 dpc embryos that lacked the endogenous gene was detectable after just 0.5 hours of staining (Fig. 2C). Importantly, no expression was detected at 14.5 dpc using the same short stain (Fig. 2D), suggesting that down-regulation of expression from the BAC transgene follows the same dynamics as the endogenous gene. After longer staining, transgenic *Gadd45g* expression could be detected at high levels at 11.5 dpc (Fig. 2E) and to a lesser degree at 14.5 dpc, in both the testis cords and Müllerian duct (Fig. 2F). No expression was detected in mutant homozygous tissue at either stage, whether with short or long staining (Fig. 2G–J). Similar observations were made using XX samples (Supplementary Information: Fig. S1).

The onset of expression from the BAC-encoded *Gadd45g* was also the same as the endogenous gene (Fig. 2K–P). No gonadal expression was detected at very early stages (6 ts, approximately 10.2 dpc)(Fig. 2K–M), but expression was detected in wild-type and transgenic embryonic gonads at 9 ts (around 10.5 dpc)(Fig. 2N,P). Expression was restricted to the centre of the gonad, as previously reported^6^, and was stronger in transgenic embryos.

**Figure 3:**
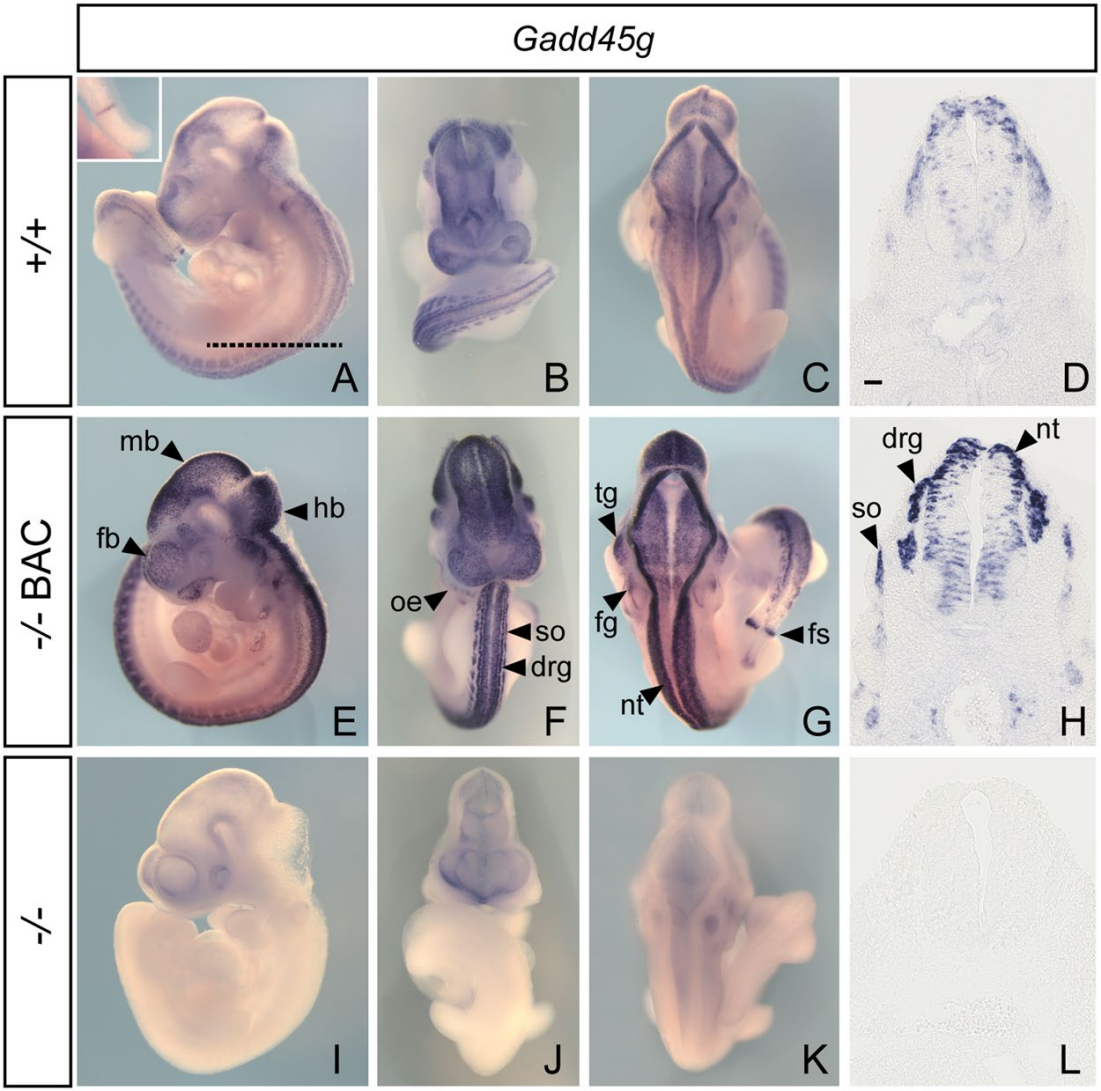
WMISH analysis of *Gadd45g* in wild-type (+/+), transgenic null (−/− BAC) and null (−/−) embryos at 10.5 dpc. Transverse sections of all three genotypes are shown in (**D**,**H**,**L**) (at the level indicated in panel (**A**). No signal is detected in null embryos (**I**–**L**). Inset in panel A shows forming somite. mb, midbrain; fb, forebrain; hb, hindbrain; oe, olfactory epithelium; so, somite; drg, dorsal root ganglion; tg, trigeminal ganglion; fg, facial ganglion; nt, neural tube; fs, forming somite. Scale bar = 50 μm.

We then determined whether extra-gonadal expression of the BAC-encoded gene matched that of the endogenous gene by examining expression in whole embryos at 10.5 dpc. *Gadd45g* expression in wild-type embryos was detected in the neural tube, the trigeminal and facial acoustic ganglia, the dorsal root ganglia, the forming tail somite, the olfactory epithelium and somites (Fig. 3A–D). These sites of expression are consistent with those previously reported^16^. All these sites were recapitulated in the presence of the transgene and in the absence of the endogenous gene (Fig. 3E–H). As predicted, levels of expression were higher in transgenic embryos. No expression was detected in homozygous knockout embryos lacking the transgene (Fig. 3I–L).

**Figure 4:**
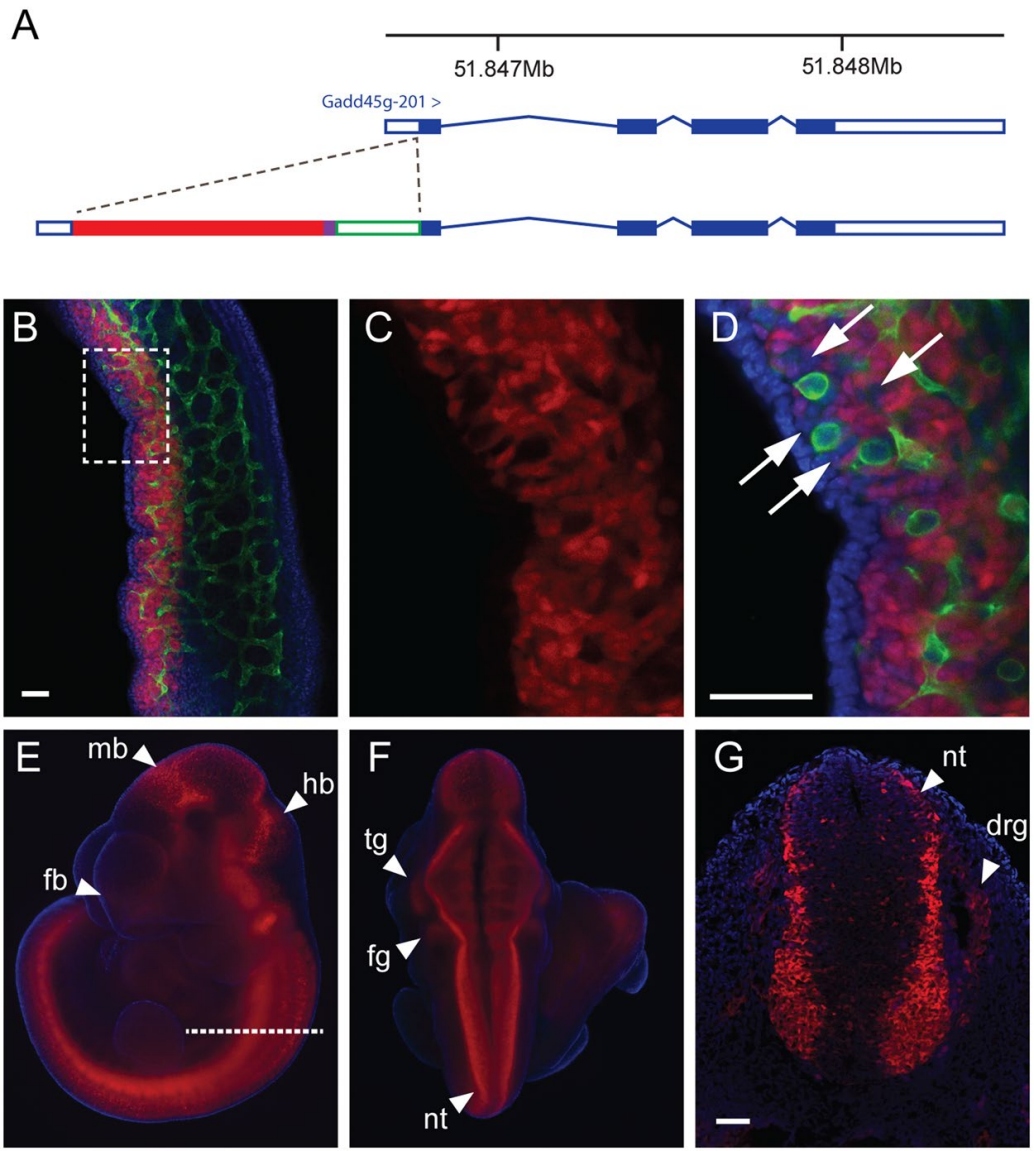
(**A**) Position of the mCherry reporter in the *Gadd45g* open reading frame of E15 BAC; (**B**) Detection of Cherry fluorescence in 11.5 dpc embryonic gonad. Green signal indicates location of PECAM. Blue is DAPI staining. (**C**) Cherry signal is detected in somatic cells of the gonad, which lack PECAM staining shown in (**D**). A few somatic cells (lacking PECAM) also lack Cherry signal (white arrows). (**E**) Lateral view of 10.5 dpc embryo showing Cherry fluorescence in developing neural tissue (forebrain (fb), midbrain (mb), hindbrain (hb)). (**F**) Dorsal view of same embryo reveals signal in neural tube (nt), trigeminal ganglion (tg) and facial ganglion (fg). (**G**) Section of embryo (in plane indicated by dotted line in (**E**)) shows neural tube and dorsal root gangion (drg) fluorescence. Scale bar = 50 μm.

Finally, as a proof of principle, we modified the E15 BAC using the Red/ET recombineering method^17^ in order to introduce a fluorescent mCherry reporter into the *Gadd45g* open reading frame after the first codon (Fig. 4A). Seven lines of founder mice were generated by microinjection of MII oocytes and these were used in timed mates to generate embryos between 10.5 and 11.5 dpc. Embryos from three founders exhibited Cherry fluorescence in the developing gonads of both XX and XY individuals (Fig. 4B–D and data not shown). Wholemount immunostaining of 11.5 dpc transgenic gonads with anti-PECAM antibody, which marks germ cells and endothelial cells, revealed Cherry localisation to most, but not all, gonadal somatic cells. Imaging of 10.5 dpc embryos revealed Cherry signal in the neural tube and other reported sites of expression at this stage (Fig. 4E–G). This reporter line can now be used for a variety of imaging and cell sorting applications.

## Discussion

We show here that the E15 BAC contains sequences that drive embryonic expression of *Gadd45g* in a pattern indistinguishable to that of the endogenous gene at the stages examined. This recapitulation extends to the temporal dynamics of *Gadd45g* expression in the gonad. The conservation of this profile suggested that E15 would make a good substrate for the generation of a reliable reporter of embryonic gonadal *Gadd45g* expression and probably other sites of expression. The *Gadd45g-*Cherry reporter described here, for example, could be used in live imaging of the behaviour of the supporting cell lineage in both XY and XX gonad development from the earliest stages, as has been performed for other lineages^18^.

BAC transgenic expression of *Gadd45g* was reliably detected at significantly higher levels to that of the endogenous gene. This increased expression did not result in any overt phenotypic abnormalities and may make it easier to detect sites of expression not easily detected in wild-type mouse embryos. However, caution should be exercised in how additional sites of expression in transgenic embryos are interpreted. For example, the Müllerian duct expression readily detected in E15 transgenic embryos at 14.5 dpc is much more difficult to detect in wild-type embryos (see Fig. 2B), but it is unclear whether this is due to the ease of detection of the elevated transcript levels of the transgenic tissue or whether occasional ectopic expression is observed, either due to missing inhibitory elements or disturbance to the transcriptional regulation of *Gadd45g* caused by the copy number of the BAC transgene. Independent verification of such novel sites would be required, but, if verified, they may also suggest that particular, additional phenotypic tests should be performed on the knockout.

The characterisation of the E15 BAC reveals it to be suitable for modification by recombineering as a means of studying the embryonic role of GADD45γ. It remains unclear as yet whether all adult sites of expression of *Gadd45g* are supported by this BAC; however, given the extensive neuronal gene expression detected in reporter mice transgenic for just 1767 bp of DNA upstream of the *Gadd45g* transcription start-site, it is likely that all the sites of expression reported by Kawaue *et al*.^11^ will be recapitulated in the E15 transgenic line. Definitively establishing that all sequences required for a particular function are present in the BAC requires rescue of the phenotype associated with loss of these sites of expression, as performed here for testis determination.

In summary, we identify here a BAC (E15) that supports functional *Gadd45g* transgenesis. All endogenous embryonic sites of expression tested appear to be conserved by expression from the BAC transgene and this clone represents a suitable tool for the generation of genetic modifications aimed at marking or tagging the gene. We describe the generation of a *Gadd45g*-mCherry reporter line using BAC recombineering that exhibits robust embryonic gonadal and extra-gonadal expression; other modifications, including the introduction of Cre recombinase or a highly efficient epitope tag, can be envisaged. Given the hypothesized role of changes to the *GADD45g* regulatory region in the evolution of human-specific traits^19^, a functional mouse *Gadd45g* transgene can be a very useful tool for further study of the regulation and function of this important gene.

## Methods

### Mouse strains and ethical approval

All mouse experimentation was approved by the Animal Welfare and Ethical Review Body (AWERB) at the MRC Harwell Institute, UK. Mice used were bred with licensed approval from the UK Home Office (PPLs 30/2877 and 70/8988). Mice were housed in individually ventilated cages (IVCs) in a specific pathogen-free (SPF) environment. Further details of micro- and macro-environmental conditions are available on request. Mice lacking *Gadd45g* have been previously described^6^. All lines were generated and maintained on C57BL/6 J (B6).

### Generation of BAC transgenic mice

The RP23–194E15 (E15) BAC was initially characterised to ensure that all *Gadd45g* exons were present and free of mutation. Microinjection of single-cell B6 embryos with E15 BAC DNA was performed as previously described^13,20^. Co-injection of metaphase II oocytes with the modified E15-Cherry BAC and sperm was performed as described^14^. The E15 transgene was detected by amplification of the chloramphenicol resistance gene (*Cmr*)^20^. In order to identify E15-positive, *Gadd45g*^−/−^ mice, *Cmr* detection was used to confirm presence of the BAC; a qPCR copycaller assay (Applied Biosystems) for *neo* was used to detect the *Gadd45g* null allele and a qPCR copycaller assay was also used to detect copies of *Gadd45g*, which were higher in the BAC transgenics. mCherry-positive mice were detected by PCR. All primer sequences are available on request.

### BAC recombineering

BAC recombineering was performed using the counter-selection BAC modification kit (Gene Bridges) according to manufacturer’s instructions. Briefly, a rpsL-neo targeting cassette was generated that included *Gadd45g* homology arms. In the first step, this was electroporated into E15-positive *E. coli* cells to confer resistance to kanamycin and sensitivity to streptomycin. A construct containing mCherry, nuclear localization signal and SV40pA terminator derived from Addgene plasmid GW1-Peredox-mCherry-NLS (#32381, a kind gift from Gary Yellen) was used to replace the rspL-neo cassette in a second round of transformation, restoring streptomycin resistance. Unmodified, rpsL-neo integrant and mCherry recomineered BAC DNA samples were digested with *Sca*I and resolved on a 0.5% agarose gel to reveal 4.35 kb, 5.8 kb and 5.3 kb fragments, respectively, confirming fidelity of the two-step modification process. All other *Sca*I bands remained unchanged, suggesting that no unwanted modifications had occurred.

### Generation of embryos

Noon on the day of the copulatory plug was counted as 0.5 dpc. Adult mice were humanely sacrificed by dislocation of the neck, confirmed by palpation, and embryos were decapitated in ice-cold, phosphate buffered saline solution. Embryos collected at 11.5 dpc were staged accurately based on the number of tail somites (ts).

### Wholemount *in situ* hybridization

Wholemount *in situ* hybridization (WMISH) analysis of embryonic tissues and probes for *Sox9* and *Stra8* have been previously described^6,21^. A *Gadd45g* probe for exons 2–4, which are absent from any transcript from the targeted allele, was generated by RT-PCR from gonadal RNA. At least three independent biological samples from a given group were analysed with a particular marker.

### Quantitative reverse transcription PCR (qRT-PCR)

Total RNA was extracted using RNeasy plus micro kit (Qiagen) from gonads separated from the mesonephros. Reverse transcription (RT) was carried out with 200 ng of total RNA using the High capacity cDNA RT kit (Applied 24 Biosystem). Quantitative RT-PCR (qRT-PCR) was performed with Fast SYBR Green Master Mix (Life technologies) on a 7500 Fast Real-Time PCR system (Applied Biosystems). RNA expression levels were normalized to those of *Hrpt1* (endogenous control) using the ΔΔCt method. At least three samples for each genotype were analysed. Primer sequences are available on request.

## Electronic supplementary material

Supplementary Figure 1

## References

[1] Barreto G (2007). Gadd45a promotes epigenetic gene activation by repair-mediated DNA demethylation. Nature.

[2] Niehrs C, Schafer A (2012). Active DNA demethylation by Gadd45 and DNA repair. Trends Cell Biol..

[3] Miyake Z, Takekawa M, Ge Q, Saito H (2007). Activation of MTK1/MEKK4 by GADD45 through induced N-C dissociation and dimerization-mediated trans autophosphorylation of the MTK1 kinase domain. Mol Cell Biol..

[4] Hoffmeyer A, Piekorz R, Moriggl R, Ihle JN (2001). Gadd45gamma is dispensable for normal mouse development and T-cell proliferation. Mol Cell Biol..

[5] Gierl MS, Gruhn WH, von Seggern A, Maltry N, Niehrs C (2012). GADD45G functions in male sex determination by promoting p38 signaling and Sry expression. Dev. Cell.

[6] Warr N (2012). Gadd45gamma and Map3k4 interactions regulate mouse testis determination via p38 MAPK-mediated control of Sry expression. Dev. Cell.

[7] Johnen H (2013). Gadd45g is essential for primary sex determination, male fertility and testis development. PLoS One.

[8] Lucas A (2015). Gadd45gamma regulates cardiomyocyte death and post-myocardial infarction left ventricular remodelling. Cardiovasc Res..

[9] Gantner ML, Hazen BC, Conkright J, Kralli A (2014). GADD45gamma regulates the thermogenic capacity of brown adipose tissue. Proc Natl Acad Sci USA.

[10] Matsunaga E, Nambu S, Oka M, Iriki A (2015). Comparative analysis of developmentally regulated expressions of Gadd45a, Gadd45b, and Gadd45g in the mouse and marmoset cerebral cortex. Neuroscience.

[11] Kawaue T (2014). Neurogenin2-d4Venus and Gadd45g-d4Venus transgenic mice: visualizing mitotic and migratory behaviors of cells committed to the neuronal lineage in the developing mammalian brain. Dev. Growth Differ..

[12] Van Keuren ML, Gavrilina GB, Filipiak WE, Zeidler MG, Saunders TL (2009). Generating transgenic mice from bacterial artificial chromosomes: transgenesis efficiency, integration and expression outcomes. Transgenic Res..

[13] Gardiner WJ, Teboul L (2009). Overexpression transgenesis in mouse: pronuclear injection. Methods Mol. Biol..

[14] Perry AC (2001). Efficient metaphase II transgenesis with different transgene archetypes. Nature Biotech..

[15] Copeland NG, Jenkins NA, Court DL (2001). Recombineering: a powerful new tool for mouse functional genomics. Nature Rev. Genet..

[16] Kaufmann Lilian T., Gierl Mathias S., Niehrs Christof (2011). Gadd45a, Gadd45b and Gadd45g expression during mouse embryonic development. Gene Expression Patterns.

[17] Wang S, Zhao Y, Leiby M, Zhu J (2009). A new positive/negative selection scheme for precise BAC recombineering. Molecular Biotechnology.

[18] Coveney D, Cool J, Oliver T, Capel B (2008). Four-dimensional analysis of vascularization during primary development of an organ, the gonad. Proc Natl Acad Sci USA.

[19] McLean CY (2011). Human-specific loss of regulatory DNA and the evolution of human-specific traits. Nature.

[20] Warr N (2014). Transgenic Expression of Map3k4 Rescues T-associated Sex Reversal (Tas) in Mice. Hum Mol Genet.

[21] Bogani D (2009). Loss of mitogen-activated protein kinase kinase kinase 4 (MAP3K4) reveals a requirement for MAPK signalling in mouse sex determination. PLoS Biol..

