# Supplementary material for: Characterisation and use of a functional Gadd45g bacterial artificial chromosome
Source: Sci Rep. 2018 Nov 23;8:17318. doi: 10.1038/s41598-018-35458-5 (PMC6251886; doi:10.1038/s41598-018-35458-5)
Supplement: Supplementary file 1 — Supplementary Figure 1 [file 41598_2018_35458_MOESM1_ESM.pdf]

Characterisation and use of a functional *Gadd45g* bacterial artificial chromosome

Nick Warr, Joel May, Lydia Teboul, Toru Suzuki, Maki Asami, Anthony C.F. Perry, Sara Wells & Andy Greenfield

Supplementary Information

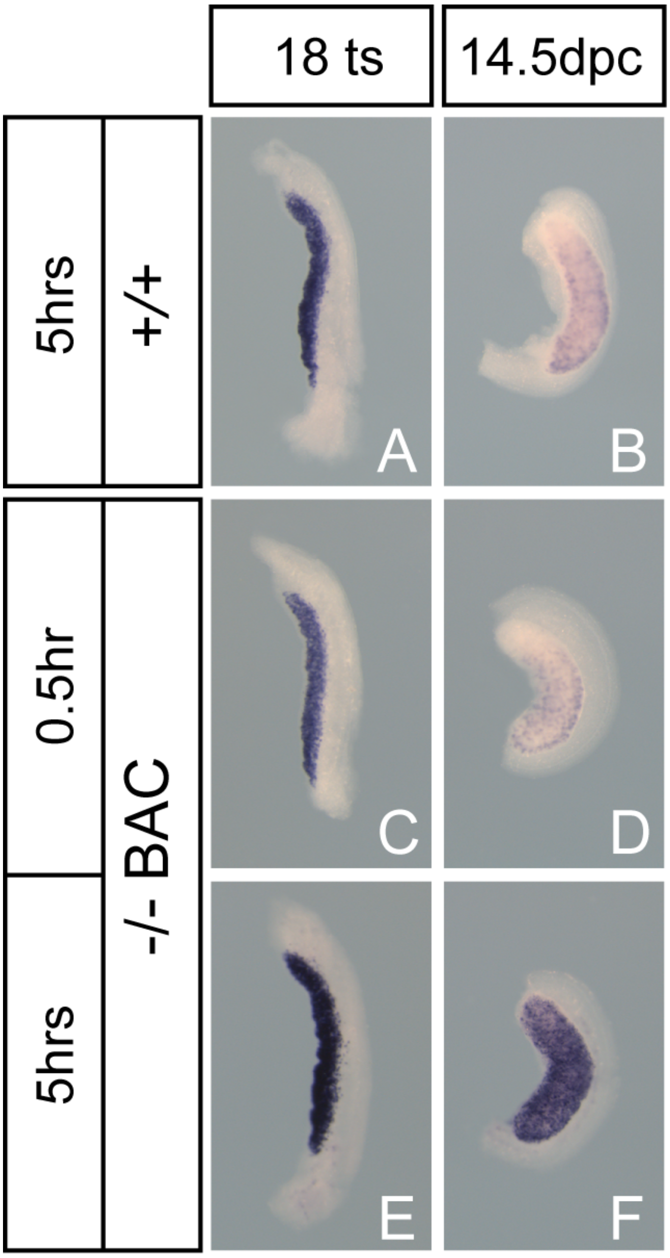

SUPPLEMENTARY FIGURE 1

Figure S1. WMISH analysis of *Gadd45g* expression in XX wild-type (+/+) at 18 ts (A) and 14.5 dpc (B). Expression is also shown for transgenic null (-/- BAC) gonads at 18 ts following staining for 0.5 hours (C) or 5 hours (E), and similarly at 14.5 dpc (D, F).
